# A review on recent studies utilizing artificial intelligence methods for solving routing challenges in wireless sensor networks

**DOI:** 10.7717/peerj-cs.1089

**Published:** 2022-10-19

**Authors:** Walid Osamy, Ahmed M. Khedr, Ahmed Salim, Amal Ibrahim Al Ali, Ahmed A. El-Sawy

**Affiliations:** 1 Computer Science Department, Faculty of Computers and Artificial Intelligence, Benha University, Benha, Egypt; 2 Unit of Scientific Research, Applied College, Qassim University, Buraydah, KSA; 3 Computer Science Department, University of Sharjah, Sharjah, United Arab Emirates; 4 Mathematics Department, Zagazig University, Zagazig, Egypt; 5 Department of Computer Science, College of Sciences and Arts, Al-methnab, Qassim University, Buridah, Al-mithnab, KSA; 6 Information Systems Department, College of Computing and Informatics University of Sharjah, Sharjah, UAE

**Keywords:** Artificial intelligence, Data aggregation, Internet of Things, Data collection, Data dissemination, Challenges, Wireless Sensor Networks, Routing

## Abstract

Wireless sensor networks (WSNs) are becoming increasingly important, providing pervasive real-time applications that have been used to enhance smart environments in various fields such as smart cities, manufacturing, and the Internet of Things (IoT). This survey reviews and analyzes the research trends related to the utilized Artificial Intelligence (AI) methods for WSN and the potential enhancement of WSNs using these methods. We highlight the routing challenge in WSN and present a comprehensive discussion on the recent studies that utilized various AI methods in addressing the routing challenge to meet specific objectives of WSN, during the span of 2010 to 2020. This would guide the reader towards an understanding of up-to-date applications of AI methods with respect to routing challenge in WSN. In addition, a general evaluation is provided along with a comparison of utilized AI methods in WSNs, which guides the reader in identifying the most appropriate AI methods that can be utilized for solving the routing challenge. Finally, we conclude the paper by stating the open research issues and new directions for future research.

## Introduction

*Ad-hoc* network technology is a growing research area that has attracted considerable attention over the years (Bellavista et al., 2013). Broadly *ad-hoc* networks can be classified into two main classes: mobile *ad-hoc* networks (MANETs) and wireless sensor networks (WSNs). Compared to MANETs, WSNs involve of low cost devices with limited power consumption (Khedr & Ramadan, 2011). Generally, MANETs are designed for mobile devices that can move freely, while WSN nodes contain embedded CPUs and smart sensors, tend to be deployed for environmental monitoring and data sensing (*e.g*., wind, air, humidity, pressure, vibration, detecting gases and chemicals, earthquake, *etc*.) (Jabbar, Saad & Ismail, 2018; Osamy et al., 2018). People’s lifestyle choices have been revolutionized by the increasing benefits of Internet of Things (IoT) based applications. In order for data to be communicated effectively, many of these IoT-based applications require an accurate identification of the node’s position and location (Bradai, Benslimane & Singh, 2015; Khedr, 2015b, 2015). The core component of IoT is the WSN, which enables many different applications and allows the dynamic connection of sensor devices to the internet (Amri et al., 2017; Khedr & Omar, 2015). Although WSNs provide valuable capabilities in areas such as science, health care, engineering, environment monitoring, home appliances, detecting forest fires, and disaster prediction, it faces different challenges due to its limited resources capabilities (Wang & Liu, 2010). In this survey, our main objective is to provide a comprehensive review of the recent studies that addressed the routing challenge utilizing various AI techniques to meet particular objectives of WSN during the period of 2010–2020. Then, a general evaluation and comparison of the used AI methods in WSNs is provided. The research community will be able to identify the most widely adapted methods to address the challenge posed by a WSN routing; and the benefits of using various AI techniques to solve the problem.

**Why it is needed:** Whilst there are many available surveys that analyze the routing challenge in WSN, this review is different from those because it aims to provide a contemporary analysis of recent literature. It reviews and investigates various AI techniques that can assist in resolving existing WSN routing problems and improve WSN performance. In addition, we provided a methodical analysis and comparison of the papers published from 2010 to 2020. This paper offers a comprehensive review of 75 related papers toward a number of fields including computer science and AI from trustworthy databases. As part of the analysis of the papers, the utilized AI techniques have been identified and classified. Initially, an overview is provided of these techniques. This paper examines the research distribution and trends that characterize AI’s role in addressing routing challenge in WSN. Moreover, the paper identifies some promising research directions in applying AI-based solutions to the WSN routing challenge, in order to facilitate and promote future research.

In summary, the following are the major contributions of this study:

1. We briefly describe the used AI techniques to overcome the routing challenge in WSNs. Then, a comprehensive discussion on the recent studies that utilized various AI methods during the span of 2010 to 2020 to meet specific objectives of addressing the routing challenge.
2. We identify promising research directions in applying AI-based solutions, with the aim to promote and facilitate further research.

**Intended Researchers:** The conducted review is intended to help the new researchers with a comprehensive discussion on the recent studies that utilized various AI methods in addressing the routing challenge to meet specific objectives of WSN during the span of 2010 to 2020. Also, the conducted survey is intended to help the researchers in the field of WSNs to look deeper for identifying promising research directions in applying AI-based solutions, with the aim to promote and facilitate further research. The methodology used to conduct this survey is discussed in the following section.

The rest of the article is structured as follows: the methodology used to conduct this survey is discussed in Section 2. Background Information discussed in Section 3. Section 4 examines related research works. In Section 5, routing challenge is discussed and corresponding AI solutions are presented. In Section 6, a discussion about the summary of the previous studies is presented. In Section 7, the respective open research issues that can guide the research community for future innovations. Section 8 provides the conclusion of the article. Table 1 summarizes the abbreviations for the key terms used in the article.

**Table 1:** Table of main abbreviations.

Abbreviation	Description
ANN	Artificial Neural Network
ABC	Artificial Bee Colony
AI	Artificial Intelligence
BS	Base Station
CH	Cluster Head
DL	Deep Learning
FL	Fuzzy Logic
HS	Harmony Search
IoT	Internet of Things
MAS	Multi-Agent System
PSO	Particle Swarm Optimization
RL	Reinforcement Learning
SI	Swarm Intelligence
WSN	Wireless Sensor Network

## Methodology

**Figure 1:**
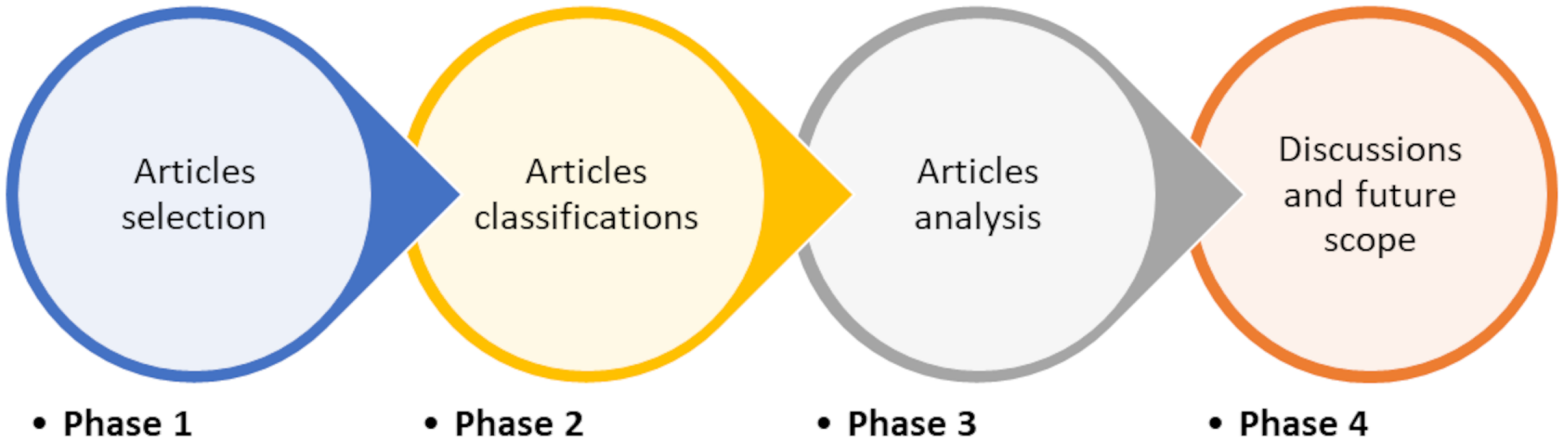
Research methodology.

In this section, we follow the same research methodology presented by Osamy et al. (2022a, 2022b). As indicated in Fig. 1, the research methodology used here is separated into four phases. The articles selection phase (Phase 1) is comprised of two steps: database source selection and article selection and filtering. The classification of articles is part of phase 2. Phase 3 entails article analysis, which is discussed further in the “AI based solutions for routing challenge in WSNs”, and Phase 4 entails discussions and future scope which is covered later in “Discussions” and “Future scope”.

### Articles selection phase

- **Database sources selection step:** The article sources and the key search strategies may effect the research quality, for that reason the included papers in this survey are selected from reliable sources and only indexed journals are considered. Table 2 shows list of sources.

**Table 2:** Database sources.

Source	URL
Scopus	https://www.scopus.com/
IEEE explore	https://ieeexplore.ieee.org/
Web of Science	https://clarivate.com/webofsciencegroup/solutions/web-of-science/
ACM digital library	https://dl.acm.org/
- **Articles selection and filtering step: **Our search query is composed of research-related terms, brief phrases, and Boolean operators (AND and OR). The whole processes of forming the query strings shown Fig. 2. Afterward, the search results are gathered and filtered, excluding those papers that are not directly related to our subject, duplicates, or of insufficient quality. Furthermore, in order to ensure that the filtered articles are eligible for our targets, we read the abstracts first and if that does not indicate eligibility, we review the content of the article. Based on that, 75 most relevant articles are selected.

**Figure 2:**
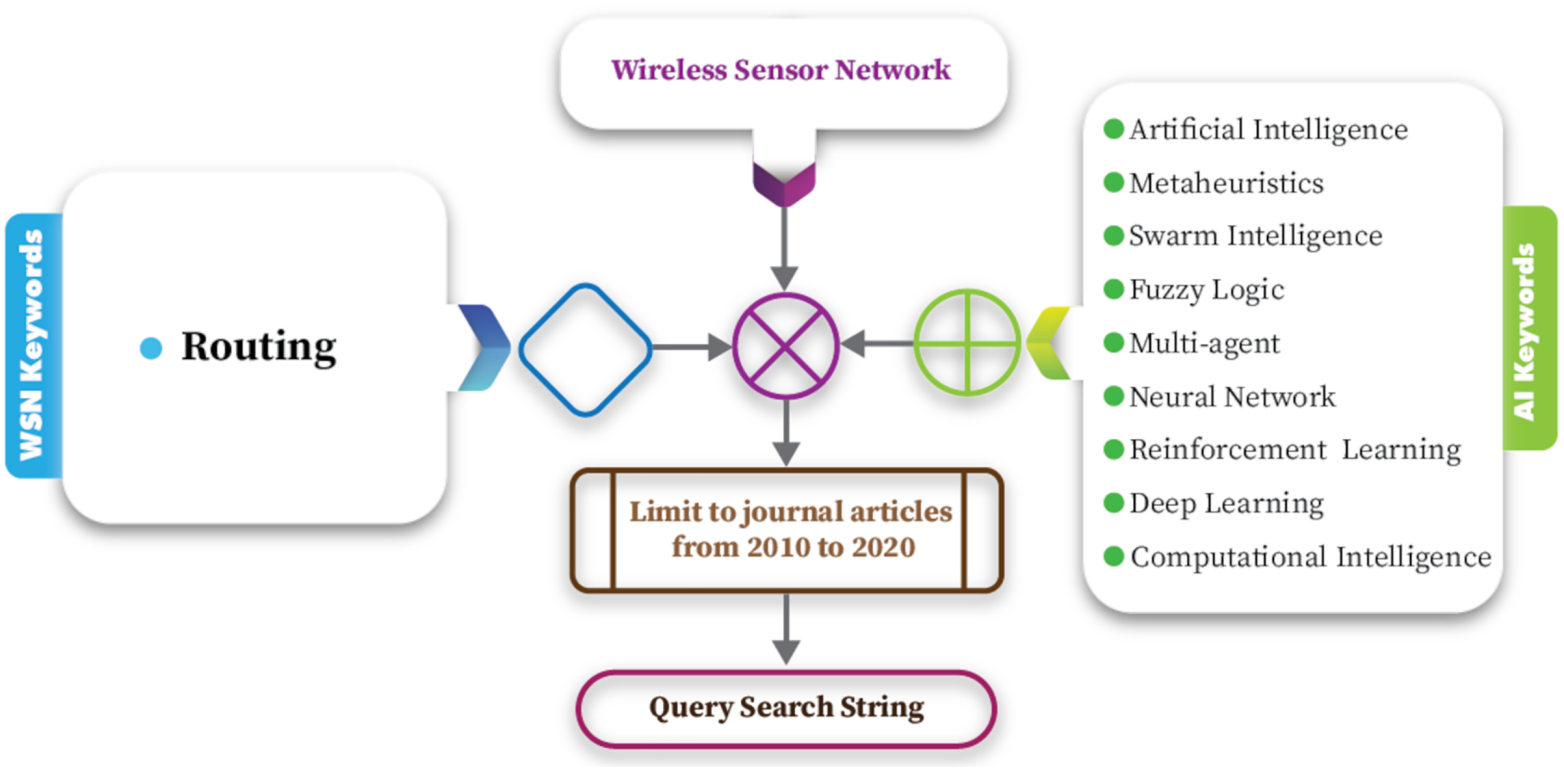
Query search string formation diagram.

### Articles classifications phase

**Figure 3:**
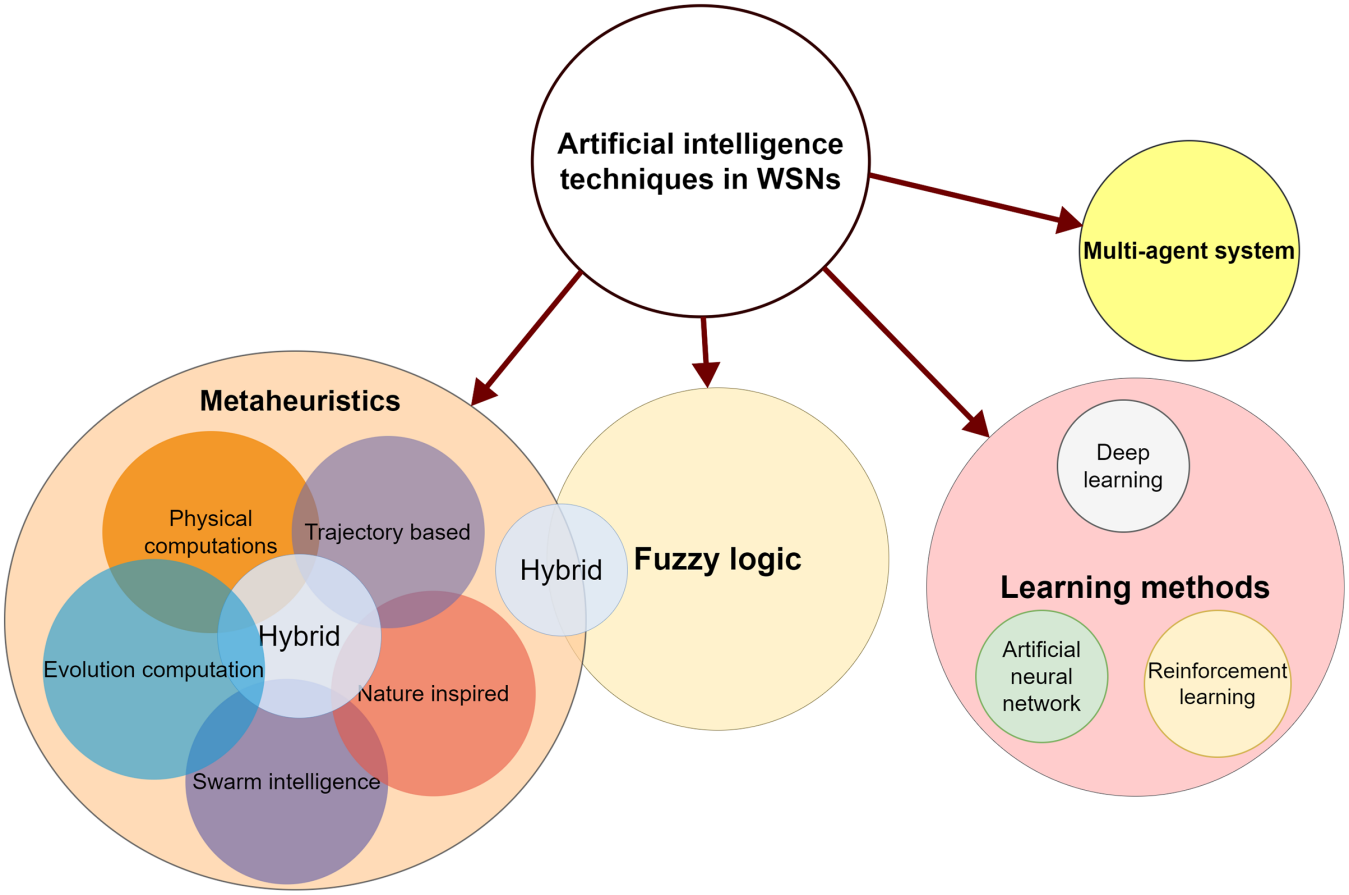
AI methods applied to address WSN routing challenge.

Among 75 papers examined, different AI techniques for routing challenge in WSNs have been identified and classified from primary database sources as shown in Fig. 3. These techniques include fuzzy logic (FL), evolutionary computation, artificial neural network (ANN), multi-agent systems (MAS), nature-inspired, trajectory based, physical computation, reinforcement learning (RL) and hybrid. An overview of these techniques is presented later in “Artificial intelligence techniques”. This classification of AI approaches is then used to demonstrate how AI strategies handled the routing issue in WSN.

## Background information

### Routing

This section provides a brief background information on the focus of our survey. WSN consists of hundreds and thousands of nodes and these nodes have the potential to communicate with each other or to the Base Station (BS) (Al-Karaki & Kamal, 2004; Raj, Khedr & Aghbari, 2020; Osamy, El-Sawy & Khedr, 2020; Khedr, 2015a; Khedr, Aziz & Osamy, 2021). Routing refers to the path that is followed by these nodes to communicate the data to its destination and it is important to have a routing strategy for convenient communication among nodes. Initially, every node is preloaded with limited capacity battery. As the data transmission progresses, the depletion of nodes energy occur. So energy conservation is the major challenge while routing in WSN. Routing protocols aim to build communication paths between the WSN nodes and the BS. Various protocols that perform routing in energy efficient manner have been developed so that they provide a uniform distribution of load between the nodes, contributing to a reduction in WSN power consumption. Each sensor node may have different routes to BS, among which the selection of optimal path is very challenging. Routing path should optimize the network lifetime. If the chosen route from a node towards BS is not optimal, it leads to increased power consumption by the network.

All sensor nodes in a WSN can monitor a specific area, and the data collected by the BS can be made available to users for further analysis (Khalifa, Khedr & Al Aghbari, 2021; Khedr & Osamy, 2012). Routing protocols are implemented to prolong the lifetime of a network since data transmission in wireless channels consumes the largest part of the total power consumed by the entire network.

Routing in WSNs is a challenging task that represents a classic trade-off between efficiency and responsiveness. To achieve the trade-off, we must take account of the limited processing and communication capabilities of sensor nodes against the overhead associated with adapting to them. In order to solve this routing challenge, we need a strategy to balance these competing interests effectively (Sohraby, Minoli & Znati, 2007). When designing routing protocols for WSNs, it is important to take into account the power and resource constraints of the network nodes, the time-varying quality of the wireless channel, as well as the possibility of packet loss and delay. Several routing strategies have been proposed for WSNs in response to these requirements. These strategies can be classified into classes. There is a class of routing protocols that employ flat network architectures, in which nodes are considered peers. Data-centric routing protocols are a class of routing protocols that use attribute-based naming, which allows a source node to query an attribute for the phenomenon rather than a specific sensor node. Location-based routing is another routing protocol class that uses a sensor node’s location to address it. This is useful when the geographic location of the sensor node is relevant to the query issued by the source node. An example of this would be specifying an area or a specific point in the network environment where an interesting phenomenon may occur. There is another class of routing protocols that impose a structured network that maximizes energy efficiency, stability, and scalability. In this class of protocols, network nodes are organized in clusters. Clustering is a classical method of extending the lifetime of a WSN by dividing all the nodes into clusters according to criteria. In each cluster, one node is chosen to be the CH (A number of metrics can be used to select the CH, including distances between the nodes and CH, distances between the nodes and BS, residual node energy, RSSI, node degree, cluster density, node weight, and position metrics) to collect the data from its CMs and send the aggregated data to the BS. By reducing the amount of data transmitted over a wireless channel, data aggregation can greatly prolong the lifetime of a network. Optimizing clustering processes in WSNs involves optimizing CH selection, data aggregation, cluster formation, and data communication. Besides, clustering protocols must be designed not only to optimize energy consumption, but also to ensure the QoS and to make sure a balance is achieved among numerous conflicting issues, such as service lifetime, coverage, and throughput (Jubair et al., 2021).

### Artificial intelligence techniques

AI refers to a computer system’s ability to accomplish activities that need human intelligence and also imitate human brains or thoughts. It is a field of computer science whose aim is to make machines smart. Among the most widely used AI techniques are search methods, learning methods, fuzzy systems, knowledge-based reasoning and fuzzy systems. AI is applicable to solve many challenges in a variety of sectors such as security, finance, health care, and transportation (Bello et al., 2015). There are various applications that utilize AI techniques along with WSNs. As an example, in agriculture, farmers can now use AI to analyze weather conditions, temperature, water usage, energy usage, and soil conditions collected on their farms through sensors in order to make better decisions. In addition to using captured sensor data in predicting yields, farmers can now use intelligent data processing techniques like machine learning to prepare for natural disasters and climate conditions. A combination of IoT-based WSN and AI is emerging as a solution toward improving agricultural productivity and efficiency (Mekonnen et al., 2019). AI implemented in Intelligent Transportation System (ITS) based WSN for energy efficient routing as well as a fast response for intra-cluster communication of the nodes to overcome the challenges for ITS (Goswami et al., 2022; Machin et al., 2018). Using AI and WSNs, we can develop a “smart pension” that can improve the quality of life for the elderly at different levels (mainly including the daily life, health care, and spiritual comfort of the elderly) (Li, Jiang & Liu, 2021). In the HABITAT (Borelli et al., 2019) project, various IoT technologies are integrated, including RFID, wearable electronics, WSNs, and AI, offering assistive and re-configurable spaces. HABITAT integrates technological solutions with the aim to enable needy people to live in their homes safely and for as long as possible, performing most of the activities related to their primary needs autonomously. ANNs and WSNs can be used for modeling applications in several domains for example, producing accurate thermal models of indoor spaces (Zhao, 2013). WHISPER (Jacoby et al., 2021) system proposed to address the energy and power demand triggered by human presence in homes. WHISPER is a maintenance-free and privacy-preserving human occupancy detection system that utilizes a local wireless network of battery-free environmental, acoustic, and image sensors to monitor homes, gather information, and transmit it to a central station. Then the BS uses many machine learning algorithms for inferring human presence from received data using hierarchical sensor fusion. AI schemes for WSN is the main focus of this survey. We have studied and classified various AI techniques used in WSN as depicted in Fig. 3. Different AI techniques used in addressing WSN routing challenge include ANN, evolutionary computation, FL, nature-inspired, MAS, trajectory based, physical computation, SI, DL, RL and hybrid. An overview of these techniques is presented as follows.

1. **Metaheuristics**
  Metaheuristics are strategies that guide the search process to efficiently explore the search space in order to find near–optimal solutions (Luke, 2013). Metaheuristic algorithms can be classified into various ways. For example, one classification consists of: trajectory-based approach which aims to find a single optimal solution (*e.g*., simulated annealing (Osamy, El-sawy & Khedr, 2019)) and population-based approach which utilize multiple solutions through search space to reach the final solution (Yang, 2010).
  Swarm Intelligence (SI) is one of the main computational intelligence methods that are used to solve complex problem. SI concerns collective study of the individuals behavior of population interact with one another in the same environment. Especially for biological systems nature often act as an inspiration (Brownlee, 2011). These techniques encourage individuals to share information and create strategies for self-organization, learning, and co-evolution during iterations in order to achieve high efficiency. Due to the simple rules the individuals follow, interactions can occur between them, and because there is no central infrastructure for demonstrating their behavior, this population can exchange related data by using any messaging system (Jagtap, 2014).
2. **Learning Methods**
  Among the most important features of humans (or animals) is their ability to learn. ‘Learning’ is the capability of acquiring new knowledge automatically and continuously, without any explicit coding. Therefore, learning forms part of AI, such as ANN, RL, and DL. ANNs have proven successful in solving complex challenges due to their ability to mimic biological neural networks and human attributes. ANNs are applied in many areas, including prediction and validation, optimization, approximation, clustering, time series analysis and pattern recognition. The literature contains several architectures of ANNs including: radial basis function networks, multilayer perceptrons (MLP), and recurrent neural networks (RNNs) (Bello et al., 2015). A branch of AI known as RL deals with how intelligent agents should operate in a given environment to maximize the concept of cumulative reward. In the RL process, learning is achieved through the interaction of learning objects with their environment. Objects seek to learn by trial and error. Based on representation learning, DL is an attractive AI function since it can learn without human supervision, using data that is both unstructured and unlabeled. DL emulates the way the brain processes data and creates patterns that can be used for decision making. DL is considered as an universal learning scheme, and it is used to solve a broad spectrum of problems in a variety of applications (Alom et al., 2019). DL differs from other machine learning approaches by representing feature extraction in multiple hierarchical levels. A variety of situations can be solved using DL that include: Expertise is difficult to explain, Over time, a problem’s solution changes, absent of human expert, and the problem size is too big for limited reasoning capabilities. Due to its widespread use, DL is frequently called the Universal Learning Technique (Alom et al., 2019).
3. **Fuzzy Logic (FL)**
  Another AI technique is FL, which mimics the way humans make decisions and is used for unreliable reasoning and incomplete information management (Bello et al., 2015). The FL system works on a ‘truth value’ of between 0 and 1 (Boucher, 2019). The fuzzy set membership can range from 0 to 1. Infuzzification, maximum and mean-of-maxima (Chen, Jakeman & Norton, 2008) are examples of fuzzy set membership.
4. **Multi-Agent System (MAS)**
  Some complex problems are difficult, or impossible, to be addressed by a single agent or a monolithic system. Multi-agent based systems play a critical role in such situations. MAS stands for self-organized intelligent system, which simulates a real-world domain with many distinct components interacting in different and complicated ways, and where system-level attributes are not easily deduced from component properties. Multiple autonomous intelligent entities known as agents make up MAS, and they are generally capable of collaborating to solve issues that are beyond the capabilities of any single member. MAS is a part of Distributed AI. Due to its inherent potential to learn and make autonomous decisions, agents solve problems and provide more flexibility. Agents use their interactions with neighboring agents or with the environment to learn new contexts and actions. Subsequently, agents use their knowledge to decide and perform an action on the environment to solve their allocated task. MASs are significant since they have been proven to have a wide range of applications (Dorri, Kanhere & Jurdak, 2018).

A detailed overview of existing research trends with respect to AI methods applied for addressing the WSN routing challenge will be presented in the following sections. We will then examine the WSN routing challenge in detail and identify various AI methods that have been adopted to address the challenge. We further present a comprehensive discussion on the recent studies regarding these techniques. Moreover, we compare them to determine suitable technique(s) in overcoming various challenging concerns regarding WSN routing and state the open research issues and future research directions.

## Related work

The research in the area of WSN has become more active over the years, and a wide range of works has been executed to enhance WSN. To address the challenges of WSN, different surveys have been conducted based on different factors (*e.g*., Osamy et al. (2022a), Majid et al. (2022), Sridhar et al. (2022) and Osamy et al. (2022b)). A survey for solving various WSN challenges using computational intelligence (CI) techniques is proposed by Kulkarni, Forster & Venayagamoorthy (2010). Under harsh and uncertain conditions with limited power supplies, some of the CI algorithms have shown to outperform, however, no real time evaluations have been done on real-life WSN scenarios, so some solutions are not at the top. Sarobin & Ganesan (2015) presents another survey regarding SI techniques in WSN. They provided some recent analyses and showed that energy efficiency can be enhanced by understanding the challenges of WSN. The survey indicates that most of the existing techniques are simulation-based and mostly focused on stationary WSNs. The survey conducted by Jabbar et al. (2013) discusses intelligent optimization of WSN using bio-inspired computing. In addition, they presented the use of hybrid approaches of CI to address some of the problems associated with non-biological systems. A variety of algorithms are employed in SI including fish school, bee colony, ACO algorithms, and PSO. SI can be used to solve routing problems as it is scalable, robust, adaptive, and distributed. Montoya, Restrepo & Ovalle (2010) focused on major topics related to WSNs, such as coverage, data fidelity, connectivity, and WSN lifetime. The authors presented AI algorithms and principles to solve the problems of WSN. Moreover, a MAS (which consists of a group of agents interacting with one another and is considered a subfield of AI referred to as distributed AI) based approach is presented. Agents sense the environment with the help of sensor devices and react to it with actuators. A survey on intelligent techniques that can be used in WSNs to minimize energy consumption was conducted by Yu (2012). AI techniques are applied over WSNs for data aggregation, redundancy reduction, and energy conservation through optimizing routing protocols.

An extensive survey on SI based routing schemes for WSN is provided by Gui et al. (2016). They compared various SI based routing protocols to show advantages and disadvantages of each technique. Most popular SI techniques discussed in this article include: ACO, BFO, PSO, ABC and many other SI techniques. They presented that the reduced overheads and real time computation features of ACO based techniques offer better performance in network routing. However, ACO has several disadvantages such as its performance is extremely dependent on previous cycle. In dynamic networks, ACO is regarded as good routing algorithm as they avoid link failures. In loosely connected node scenarios, PSO is regarded as not beneficial. ABC possess limitations such as low convergence speed when compared to others. Therefore, hybrid versions are expected to be devised by researchers. They also introduced a cluster based approach for routing in WSN using Spider Monkey Optimization (SMO). Time and space complexity is an issue in this technique. An SMO-based energy efficient routing protocol is mentioned as a future approach.

Maksimović, Vujović & Milošević (2014) presented a survey related to the use of FL in WSN. FL is considered as a promising approach to evaluate diverse parameters in an efficient manner. It improves decision making, reduces resource consumption and is suitable for resolving WSN issues such as routing, data aggregation, security, localization as well as deployment. FL can tolerate unreliable or imprecise readings easily. They presented it to be an easier and efficient technique. The disadvantage of using this technique is that the rules count grows exponentially with the variables count and storage requires much extra memory. They showed that the FL approach can solve the shortcomings of most of the algorithms. As this is a rule based approach and due to constant traversal of rules, it may slow down the event detection and decision making process. To solve this problem they also presented rule based reduction techniques which are efficient, but none of them can be considered as a general solution. A key property that must be kept in mind is that the reduction techniques employed should not affect the application accuracy. So, selection of the best reduction technique is very challenging. Future work that needs to be done is to implement a software based FL to enhance the speed of the system.

Lately, the survey presented by Al Aghbari et al. (2019) discusses the most recent AI optimization techniques to solve the routing challenge in WSNs. Techniques for optimization are examined to demonstrate which strategy will do a better job. They show that the mostly used techniques in WSNs are PSO, BFO, ABC, ACO, GA, and Firefly Algorithm.

This survey differs from others where it aims to provide a contemporary study that compares different AI techniques that can be used to seek new strategies for solving existing routing issues in WSNs and enhancing the performance of WSNs, besides it cover several techniques. The purpose of the survey is to provide a systematic review and comparison of AI schemes for routing in WSNs while covering all its aspects. To do this, we reviewed the related papers from 2010 to 2020 to comply with the standards for systematic reviews and comparisons. We outlined several open research questions that need to be addressed in the future. With a rich bibliography, this article will serve as valuable insight into current trends in WSN research and foster a commitment to new research on routing in WSNs in the future.

## Ai based solutions for routing challenge in wsns

Energy, storage, communication, and processing capacity constraints pose a major challenge to routing in WSNs. Consequently, routing schemes for WSNs should have low complexity, low communication overhead, and they should also be performant, scalable, and support prolonged operation. Table 3 summarizes the AI based solutions for routing challenge in WSN. In WSNs, a number of AI techniques are used to handle routing challenge. We describe these techniques in the following way:

**Table 3:** Summary of AI based solutions to WSN routing challenge surveyed in “AI based solutions for routing challenge in WSNs”.

AI paradigms	Algorithm	Ref.	Objective	Simulation/Real-deployment	Centralized/Distributed	Mobility	Performance metrics
SI	BeeSensor	Saleem, Ullah & Farooq (2012)	Designing efficient routing protocol	Simulation using MannaSim	Distributed	Static	Packet-delivery ratio, Latency, Energy efficiency, Control overhead, Network Lifetime.
SI	EPMS	Wang et al. (2017)	Maximizing network lifetime	Real deployment	Distributed	Static with MS	Energy Consumption, Alive Nodes, and Average delivery delay.
SI	GWO	Lipare, Edla & Kuppili (2019)	Balance energy consumption and load	Simulation using MATLAB	Centralized	Static	First gateway die, First node die, Half nodes alive, and Standard deviation of load.
SI	SICROA	Shin & Lee (2020)	Improve routing performance	Simulation using NS2	Distributed	Mobile	End-to-end delay, Packet delivery ratio.
SI	PSO	Azharuddin & Jana (2016)	Maximizing network lifetime	Simulation using MATLAB	Distributed	Static	Network lifetime and Energy consumption.
SI	Termite-Hill	Zungeru, Ang & Seng (2012)	Balancing traffic Load and network lifetime	Simulation using RMASE	Distributed	Static with MS	Throughput, Energy consumption, and Network lifetime.
SI	ATDBMA	Senthilkumar et al. (2011)	Electing CH	Simulation using SenSor simulator	Centralized	Static	Number of re-election, Average remaining energy, Data delivery ratio, Percentage of active nodes, and Network effectiveness.
SI	*i*ABC	Mann & Singh (2017b)	Optimizing energy efficiency of a network	Simulation using NS2	Centralized	Static	Energy consumption, Packet delivery ratio, WSN lifetime, Throughput.
SI	IABCP	Zhang et al. (2020)	Resolve the issue of uneven network load and excessive energy usage	Simulation using MATLAB	Centralized	Static with MS	Network life cycle, Dead nodes, Average remaining energy, First node dead, and Average single-hop distance.
SI	PSO	Yang et al. (2016)	Decrease the control overhead and minimize energy consumption	Simulation using MATLAB	Distributed	Static with MS	End-to-End Delay, Packet Delivery Ratio, Energy Consumption.
SI	PSO	Wang et al. (2019)	Avoid energy holes	Simulation using MATLAB	Distributed	Static with MS	Energy consumption, Network lifetime, and Average number of hop.
SI	PUDCRP	Ruan & Huang (2019)	Balance the energy consumption	Simulation using MATLAB	Distributed	Static	Residual energy, Surviving nodes, and Number of packets received by the BS.
SI	GMDPSO	Yang, Liu & Cao (2017)	Enhance the network performance	Simulation using MATLAB	Distributed	Static	Convergence, Average number of alive nodes, First and Last node die, Average number of unClustered nodes, and Throughput.
SI	PSO	Gao, Luo & Ma (2019)	Effectively reduce energy consumption	Simulation using MATLAB	Centralized	Static	Network lifetime, Energy consumption, Inactive sensors, and Packets received by BS.
SI	PSO	Panigrahi et al. (2013)	Reduces the computational burden on individual nodes	Simulation using MATLAB	Distributed	Static	Probability of Resolution and RMSE.
SI	PSO	Shankar, Shanmugavel & Rajesh (2016)	Improves the lifetime of sensor nodes	Simulation using MATLAB	Centralized	Mobile (quasi-stationary)	Remaining energy and Standard deviation, Alive and Dead nodes, Throughput.
SI	ESO-LEACH	Nigam & Dabas (2018)	Enhances the network’s life span	Simulation using Python	Distributed	Static	Energy dissipation and Network lifetime.
SI	TPSO-CR	Elhabyan & Yagoub (2015)	Energy efficiency, network coverage	Simulation using OMNeT++ platform	Centralized	Static	Average number of non-clustered nodes, Throughput and Average consumed energy.
SI	GAs	Obaidy & Ayesh (2015)	Minimize the communication distance in a sensor	Simulation	Distributed	Mobile	Number of heads and Total distance.
SI	PSO	Li et al. (2012)	Hierarchical clustering	Simulation	Centralized	Static	Receiving rate, Average delay of packets transmission and Energy consumption.
SI	DCH-NPSO	Ma & Xu (2015)	Energy efficient utilization	Simulation	Distributed	Static	Energy consumption and Network Lifetime.
SI	PSO	Zhou, Wang & Xiang (2016)	Prolong network longevity	Simulation using MATLAB	Distributed	Static	Energy consumption and Network Lifetime.
SI	PSO E-OEERP	RejinaParvin & Vasanthanayaki (2015)	Energy Efficient	Simulation using NS2.32	Distributed	Static	Load Balancing Ratio, Energy Consumption, Packet Delivery Ratio, Throughput, and Network Lifetime.
SI	Bee Colony (MOFPL)	Kumar & Kumar (2016b)	Maximizing the lifetime of sensor nodes	Simulation using Matlab	Centralized	Static	Network Lifetime and Energy Consumption.
SI	Bee Colony (iABC)	Mann & Singh (2019)	Energy efficient	Simulation using NS2	Centralized	Static	Throughput, Packet delivery ratio, Energy efficiency, WSN lifetime.
SI	Bee Colony (DSABC)	Panda (2018)	Improve the lifetime of the network	Simulation using object oriented C++	Distributed	Static	Average remaining energy and Standard deviation, and Count of data packets received.
SI	Bee Colony	Wang, Chen & Dong (2016)	Energy consumption balance	Simulation using MATLAB	Distributed	Static	Energy consumption, Amount of survival nodes, and Network reliability.
SI	Bee Colony (CDABC)	Masdari, Barshande & Ozdemir (2019)	Reducing the energy costs	Simulation using OMNeT++	Distributed	Static	Alive nodes, First Node Die, Inactive/dead nodes, and Network lifetime.
SI	Bee Colony	Mann & Singh (2017a)	Increase network life	Simulation using Java based platform	Centralized	Static	Packet Delivery Ratio, Average Energy Consumed, Total Packet Delivered, Throughput, and Network Lifetime.
SI	Bee Colony	Cai et al. (2015)	Energy-efficient	Simulation using Java	Centralized	Static	Packet delivery rate, Control overhead, Energy standard deviation, Energy consumption, Routing building time, and Latency.
SI	Ant Colony	Zhang et al. (2017)	Secure routing algorithm	Simulation using MATLAB	Centralized	Static	Packet Delivery Ratio, Average Energy Consumption, Packet Loss Rate, and Number of Received Packets.
SI	Ant Colony	Jiang & Zheng (2018)	Balance energy consumption	Simulation using C++	Centralized	Static	Energy consumption and Network Lifetime.
SI	MOPSO	Chaudhry, Tapaswi & Kumar (2019)	Solve multicast routing problem	Simulation	Centralized	Static	Overall Nondominated Vector Generation, Inverted Generational Distance, Hyper Volume power consumption, Average delay, Delay jitter, and Packet loss.
SI	Ant Colony	Shamsan Saleh et al. (2012)	Reduce energy usage	Simulation using QualNet	Centralized	Static	Energy consumption, Average Energy, Standard deviation of energy, and Throughput.
SI	Ant Colony	Arora, Sharma & Sachdeva (2020, 2019)	Reduce energy usage	Computer-based test bed	Distributed	Static	Network life span and Energy depletion.
SI	Ant Colony	Zungeru et al. (2013)	Reduce energy consumption	Simulation using MATLAB	Centralized	Static	Energy Consumption, Latency, Success Rate, Standard Deviation, and Lifetime Prediction.
SI	Honey Bees ABC-SD	Ari et al. (2016)	Energy efficient	Simulation using Castalia 3.2	Centralized	Static	Network lifetime, Energy consumption, Energy efficiency, First sensor dead, Throughput, and Network coverage.
SI	Glowworm UCRA-GSO	Yu et al. (2019)	Balance energy	Simulation using MATLAB	Centralized	Static	Network lifetime and Residual energy.
FL	FL-EEC/D	Hamzah et al. (2019)	Reduce energy consumption	Simulation using. NET FuzzyLite library	Centralized	Static	Network lifetime and Remaining energy.
FL	FPCA	Akila & Venkatesan (2016)	Prolong network lifetime	Simulation using NS2	Centralized	Static	Average PDR, Average E2E delay, Control overhead, and Average packet drop.
FL	AGRR	Zhang et al. (2018)	Reduce energy consumption	Simulation using NS2	Centralized	Static	Network reliability, Maximum energy cost, and Maximum E2E delay.
FL	FCM-3 WSN	Hai, Son & Vinh (2017)	Reduce energy consumption	Simulation using C	Centralized	Static	Energy consumptions and Network lifetime
FL	NSGA-II	Sert, Bagci & Yazici (2015)	Reduce energy consumption	Simulation	Centralized	Static	Network lifetime, Number of packets arrived to Sink, and Total energy.
FL	LEACH-SF	Shokouhifar & Jalali (2017)	Prolong network lifetime	Simulation	Centralized	Static	Intra-cluster distances, WSN lifetime, Number of received data packets in BS, CPU running time.
FL	EUDFC	Yuste-Delgado, Cuevas-Martinez & Triviño-Cabrera (2019)	Prolong network lifetime	Simulation using MATLAB	Distributed	Static	Network lifetime.
FL	FIS	Razzaq & Shin (2019)	Maximize energy-efficiency	Simulation using MATLAB	Centralized	Static	Network lifetime, Residual Energy, Delay, and Throughput.
FL	FIS	Kiran, Smys & Bindhu (2020)	Prolong the stability period	Simulation	Distributed	Static	Energy consumption.
FL	FLEC	Verma et al. (2020)	Extend the network lifetime	Simulation using MATLAB	Distributed	Static	Network stability (first node dies), half-alive nodes, network lifespan, latency, throughput, and residual energy of nodes
Evolutionary computation	DECA	Kuila & Jana (2014)	Prolonging network lifetime	Simulation using MATLAB	Centralized	Static	Network lifetime, Last gateway die, and Energy Consumption.
Evolutionary Computation	GA	Ragavan & Ramasamy (2018)	Reduce energy consumption	Simulation using NS2	Centralized	Static	Energy consumption and Delivery ratio.
Evolutionary Computation	GA (GECR)	Wang et al. (2018)	Improve energy efficiency	Simulation using MATLAB	Centralized	Static	ALive nodes and Energy consumption.
Physical Computations	Gravitational Search	Morsy, AbdelHay & Kishk (2018)	Prolong network lifetime	Simulation	Centralized	Static	Average Energy Consumption, Dead Nodes Percentage, Number of CHs per round, and Number of data packets sent to BS.
Physical Computations	Optic Inspired Optimization (CRWO)	Lalwani, Banka & Kumar (2017)	Reduce energy consumption	Simulation using MATLAB	Centralized	Static	Residual energy, WSN lifetime, Convergence rate, Received packets at the BS.
Physical Computations	iASEF	Dohare & Singh (2020)	Prolong network lifetime and optimal routing	Simulation using MATLAB	Distributed	Static	Network lifetime, Alive nodes, Dead nodes, Stability period, and Residual energy.
Trajectory based	Tabu Search (TSRA)	Orojloo & Haghighat (2016)	Reduce energy consumption	Simulation using MATLAB	Centralized	Mobile	Average energy consumption, Average network lifetime, and Average length of paths.
Nature Inspired	BAT	Cai et al. (2020)	Optimize elections in CHs	Simulation using MATLAB	Distributed	Static	Number of surviving nodes and Residual energy.
Nature Inspired	Multiparticle Swarm Immune (MPSICA)	Ding et al. (2015)	Reduce energy consumption	Simulation using MATLAB	Centralized	Static	Energy depletion ratio, Nodes survival ratio, Packet delivery ratio, and Delay of packet.
Nature Inspired	HS	Singh & Sharma (2018)	Network lifetime	Simulation using MATLAB	Centralized	Static	Dead CHs, Dead sensor nodes, Total energy consumption, Network lifetime, and Packets count received by BS.
Nature Inspired	HS	Lalwani et al. (2018)	Increase number of alive Nodes	Simulation using MATLAB	Centralized	Static	Alive Nodes count, Remaining energy, Number of data Packets received at BS.
Nature Inspired	HS (IHSBEER)	Zeng & Dong (2016)	Maximizing the network lifetime	Simulation using C++	Centralized	Static	Average Remaining Energy, WSN Lifetime.
Nature Inspired	HS (IHSBEER)	Zeng et al. (2017)	Maximize energy efficiency	Simulation using C++	Centralized	Static	Network lifetime, Active regular nodes, and Energy consumption.
Nature Inspired	BAT	Cui et al. (2019)	Increase the number of alive nodes	CEC2013 test suit	Centralized	Mobile	Network lifetime and Energy consumption.
Nature Inspired	BAT (cBA)	Nguyen, Pan & Kien (2019)	Uneven clustering problem	Simulation	Centralized	Static	Alive nodes, Similarity measure, and Time complexity.
A Hybrid of ABC and ACO	PSO-HSA	Kumar & Kumar (2016a)	Improving network lifetime	Simulation	Distributed	Static	Network lifetime, Standard deviation of residual energy, and Average Residual Energy.
A Hybrid of GWO and PSO	IPSO–GWO	Elhoseny et al. (2020)	Maximize energy efficiency and network lifetime	Simulation	Distributed	Static	Network lifetime, Packets to BS, and Average energy consumption.
A Hybrid of Fuzzy and Flower Pollination Algorithm	EFPA	Mina et al. (2020)	Prolong performance parameters of WSN	Simulation using MATLAB	Distributed	Static	Network lifetime, Residual energy, and Total energy consumption.
A Hybrid of HS and PSO	PSO-HSA	Anand & Pandey (2017)	CH selection and path selection	Simulation	Centralized	Static	Network lifetime, Standard deviation of residual energy, and Average Residual Energy.
A Hybrid of Bacterial Foraging and fuzzy	Hybrid BFO	Sindhuja & Ramamoorthy (2017)	Improving network capacity	Simulation using OPNET and MATLAB	Centralized	Static	Average end to end delay, packet delivery rate, average remaining energy at half node dead.
A Hybrid of Gravitational search and Fuzzy	Gravitational search (GSA-EEC)	Tomar & Shukla (2019)	Prolong network lifetime	Simulation using C	Centralized	Static	Delivery ratio, Packet drop, Packet delay, Throughput, Overhead, and Energy consumption.
A hybrid of Shuffled Frog Leaping Algorithm and FL	FMSFLA	Fanian & Rafsanjani (2020)	Extend network lifetime and protocol scalability	Simulation using MATLAB	Distributed	Static	WSN lifetime, Obtained data packets count at BS, Mean number of CHs.
A hybrid of Shuffled Frog Leaping Algorithm and Firefly Algorithm	MOSFA	Barzin et al. (2020)	Prolong network lifetime	Simulation using MATLAB	Distributed	Static	Energy Efficiency, WSN lifetime and Average of successfully received data packets by the sink.
DL	Artificial agent	Lu et al. (2019)	Minimizing energy consumed	Simulation	centralized	Static	Energy consumption.
RL	QLRR-WA	Künzel et al. (2020)	Enhance network performance	Simulation using NS2	Centralized	Static	Network lifetime.
ANN	ELDC	Mehmood et al. (2020)	Improve network lifetime	Simulation using OMNET	Distributed	Static	Energy Consumption and Alive nodes.

Hierarchical cluster-based routing is considered as a common communication strategy in WSNs. However, if it is not handled properly, it could rapidly deplete the resources of CHs, requiring periodic reelection. Repeated elections for CH lead to higher advertising messages and, as a result, lower energy efficiency for the WSN.

### Solutions based Swarm Intelligence

Senthilkumar et al. (2011) proposed ATDBMA (advertisement timeout driven Bee’s mating algorithm) to reduce communication overhead when creating clusters. A honeybee mating algorithm is used in selecting a standby CH in advance which can survive several rounds. By doing so, the required number of re-elections is reduced and the energy level between rounds remains reasonable.

Multipath routing is also a well-known routing technique that simultaneously use multiple alternative paths through a WSN. The authors Zungeru, Ang & Seng (2012) developed a Multipath routing algorithm that imitates the real termites behavior in hill building and is termed as Termite-Hill algorithm that supports sink mobility. In the proposed scheme, termite agents are modelled to match the energy constraints in WSN. The objective is to estimate the best path for effectively relaying the data as a function defined by considering both energy as well as visited nodes count in the path. Sink mobility is applied in this approach to balance energy usage, avoid energy holes and hot spot problem in a network which results in extended network lifetime. This is a probabilistic on-demand routing algorithm that is inspired from hill building real termites. Termite agents correspond to packets that are moving in a network and changes their routing information to locate the best route towards the termite hill and hill in this correspond to sink node in WSN. This approach balances the network reliability with the lifetime, and still have better throughput and WSN lifetime. The results of simulation reveal that it balances the energy and improves the WSN lifetime with an improvement in reliability of the network.

A GWO based technique for energy efficient cluster formation and routing in WSN is applied by Lipare, Edla & Kuppili (2019) with the objectives of conserving energy and preventing accidental failures due to battery drain.

Particle swarm optimization (PSO) is a population-focused stochastic search method inspired by the social behavior of birds flocking and fish schooling. The simple implementation, clear mechanism and quick convergence properties make PSO a widely applicable technique for solving many optimization problems. PSO is used in solving the WSN routing challenge, in many works, for example, Li et al. (2012), Panigrahi et al. (2013), Elhabyan & Yagoub (2015), Obaidy & Ayesh (2015), Ma & Xu (2015), RejinaParvin & Vasanthanayaki (2015), Yang et al. (2016), Zhou, Wang & Xiang (2016), Yang, Liu & cao (2017), Wang et al. (2019), Ruan & Huang (2019), Nigam & Dabas (2018), Gao, Luo & Ma (2019).

Li et al. (2012) used the enhanced PSO for hierarchical non-uniform clustering where multi-paths are created between the adjacent hierarchical nodes using the best-worst ant scheme. The maximum likelihood method and diffusion PSO (DPSO) algorithm were applied for direction-of-arrival estimation of clustered WSN (Panigrahi et al., 2013). However, the computational complexity is high, making it inappropriate for WSN with constrained resources. Elhabyan & Yagoub (2015) proposed a PSO-based scheme for routing in clustered WSN. In the clustering phase, the fitness function, which comprises of energy, quality cluster, and WSN coverage, is defined. The fitness function for routing stage is formulated using energy and connection quality. It does not, however, take account of the power control in the formulated fitness function. In RejinaParvin & Vasanthanayaki (2015), cluster formation and CH selection are done using the PSO algorithm. During clustering, special care is needed to avoid the creation of separate nodes. To avoid the development of residual nodes, the average clustering ratio is computed. In Ma & Xu (2015), an energy distance aware cluster oriented routing mechanism with Dual CHs utilizing Niching PSO (DCH-NPSO) is presented. DCH-NPSO selects MCH and SCH in a cluster using Niching PSO, taking into consideration parameters such as remaining energy and distance to balance and reduce energy usage.

Another PSO based scheme in combination with GA is presented by Obaidy & Ayesh (2015). They suggested a hybrid method based on an intelligent GA-PSO to extend the lifetime of mobile WSNs. GA is used in this protocol to build optimal clusters that decrease network energy consumption by reducing the distance between nodes and the BS. PSO is utilized to offer distance control, allowing the WSN to function independently. The results of simulations demonstrate that this method successfully reduces the distance in mobile WSNs.

The enhanced PSO technique, which is an optimization approach developed to choose target nodes (Zhou, Wang & Xiang, 2016), is used to extend the network lifespan. Relay nodes are utilized to ease the CHs’ high power consumption, and the protocol takes into consideration both energy efficiency and transmission distance.

An effective routing approach that is devised as a problem of optimization and uses the algorithm of PSO to construct the optimal routing paths (Yang et al., 2016). But it focuses only on addressing the regular shift from source nodes to sinks induced by routes, and did not give considerable importance in forming efficient clusters. Greedy discrete PSO with memory (GMDPSO) proposed by Yang, Liu & cao (2017) is an improved version of discrete PSO by Yang et al. (2016). In GMDPSO, under a discrete case, particle location and velocity are redefined; particle update conditions are reconsidered based on WSN topology. In addition, a greedy search technique is utilized to guide particles to rapidly locate a better location by optimizing greedy forwarding routing. In addition, search history is memorized to speed up convergence.

A PSO based technique is developed by Azharuddin & Jana (2016) for providing better solution to cluster based routing problem in large-scale WSNs. In this scheme, they addressed the hot spot problems by using unequal clustering and offered a balanced load distribution for the CHs close to the BS. PSO is a very efficient algorithm for finding optimal solution in less time. They introduced a suitable fitness function for routing and proposed a linear programming solution for clustering and routing problem. In clustering phase, unequal cluster radius for a CH is computed depending on its lifetime estimated during the routing phase. Moreover, they improved the CH lifetime by allocating only fewer nodes. Extensive simulations reveal that the technique shows better performance in WSN lifetime, active nodes count, energy efficiency and packet delivery ratio. In Wang et al. (2017), a PSO based routing technique with MS (termed as EPMS) is proposed for improving the energy efficiency of WSN. In this algorithm, virtual clustering concept is applied in the routing process. The PSO based clustering is performed to convert the WSN into distinct regions. Then, the CH for each region is selected using the location and remaining energy of nodes as key parameters. EPMS uses a well designed control schedule for MS to gather data from CHs. Three types of data packets are defined: Hello, Message-s and Message-h packets. Hello packet helps to identify the cluster area from where the data is sent to the MS. Message-h indicates that the information is to the CH and Message-s denotes the data to sink node. Simulation results indicate that the EPMS approach provides balanced energy consumption, minimizes the delay in transmission and enhances the WSN lifetime.

A special clustering scheme named energy centers searching based on PSO (EC-PSO) is discussed by Wang et al. (2019). First, the protocol selects CHs by dividing the location of the nodes according to geometric methods. PSO is then used to scan the network’s energy center, and the selected CH is the node that is closest to the energy center. Finally, in order to prevent weaker nodes being relay nodes, the algorithm suggests a protection strategy with low-energy expenditure. By integrating the above approach, the protocol will essentially prolong the lifespan of the network. As it employs a geometric technique to achieve equal distribution of CHs during the initial run, EC-PSO is only suitable to network settings with uniformly dispersed nodes.

In Ruan & Huang (2019), the authors used a PSO-based approach and presented a PSO-based uneven dynamic clustered multi-hop routing mechanism (PUDCRP). This method used an adaptive clustering strategy to help balance each node’s energy usage. In addition, a connecting line-assisted route construction technique is given for selecting a suitable node as the data communication’s next hop. Two elements influence the fitness function: coverage rate and intersection over universal (IoU) (the ratio of the candidate CH nodes count in the overlapping part in circular area to another circular area to the total count of nodes in WSN).

In Yu et al. (2019), another technique for dealing with uneven clustering in WSN is presented. To tackle the WSN energy balancing problem, an uneven clustering routing method based on the Glowworm Swarm Optimization for WSNs (UCRA-GSO) is suggested. To identify the best clustering technique, UCRA-GSO incorporates variables including CH density, CH nearness distance, CH energy, and cluster compactness into a GSO algorithm.

In Chaudhry, Tapaswi & Kumar (2019), an improved technique known as Forwarding Zone (FZ) enabled MOPSO (FZMOPSO) is proposed to overcome the limitations of conventional Multi-Objective PSO (MOPSO) in solving the multicast routing problem in WSN. Instead of iterative searching as in MOPSO, the FZMOPSO searches only in FZ for a valid solution. This greatly decreases energy and computational power relative to MOPSO. In addition, they proposed and incorporated FZ based Valid Particle Generator (FZVPG) operator with FZMOPSO to effectively turn invalid particles into valid ones.

The work by Ding et al. (2015) developed a multi-particle-swarm immune cooperative method to offer intelligent routing recovery (MPSICA). The MPSICA may save the K disjoint routes from each source node to the nearest supernode, as well as the potential supernode-to-sink path. It should also look into the best alternate routing techniques and solve the issues with cloning, high-frequency mutations, and clone selection processes, which may enhance fault handling and data transmission efficiency between clusters and within clusters. The broken path of the intercluster supernodes or the intracluster network of ordinary nodes may be efficiently restored using this technique. Finally, performance analysis experiments are used to explain and assess the implementation of the MPSICA-based method for fault-tolerant routing Elhoseny et al. (2020) proposes a new SI-based clustering and multihop routing system for WSN. Initially, an enhanced PSO approach is used to choose the CHs and efficiently structure the clusters. The grey wolf optimization (GWO) algorithm-based routing technique is then used to determine the network’s best routes. This improved PSO-GWO technique, which incorporates the benefits of both clustering and routing strategies, results in optimal energy usage and WSN lifespan.

Shamsan Saleh et al. (2012) proposed a self-optimizing technique for energy balanced routing in WSNs utilizing ants to enhance network performance. Zungeru et al. (2013) suggested an ant-based routing method for maximizing energy efficiency.

A multi-attribute pheromone ant secure routing approach (MPASR) that relies on reputation value was developed by Zhang et al. (2017). By screening nodes with greater coincidence rates and refining the technique used to update the nodes’ communication behaviors, this approach can minimize WSN energy consumption and enhance the reliability of the nodes’ reputations. Simultaneously, the node reputation value, remaining node energy, and transmission latency are combined to produce a synthetic pheromone, which is utilized in the formula for computing the random proportion rule in classic ant-colony optimization to choose the best path for data transmission.

Jiang & Zheng (2018) presented a hybrid routing method that combines ACO and a minimal hop count approach. The suggested method can discover the best route with the least amount of overall energy consumption and balanced energy consumption on each node. With the goal of leveraging the benefits of the ACO algorithm, the swarm-intelligence-centric routing algorithm (SICROA) by Shin & Lee (2020) is proposed for usage in WSNs. Through collision avoidance, link-quality forecasting, and maintenance methods, the study overcomes the issues of the adhoc on-demand distance vector (AODV) and improves routing performance.

Saleem, Ullah & Farooq (2012) proposed BeeSensor protocol which is an event driven, reactive and on demand multi path routing method for WSN. The protocol mimics the foraging characteristics of honey-bees and considers the pertinent features of *BeeHive* and *BeeAdHoc* protocols. For protocol design, three phases are included: (1) They first developed a simple, decentralized and distributed protocol for routing in WSN by taking inspiration from biological systems. (2) Then, they formally modeled the key performance metrics to obtain an analytic insight into the behavior of the proposed protocol. (3) Then, in the third phase, the protocol is improved based on the analysis results from the preceding phase. BeeSensor consists of four distinct phases: (1) scouting, (2) foraging, (3) swarming and routing loops, (4) path maintenance. This protocol is more efficient, energy aware and scalable when compared to other protocols and requires only less communication and processing cost. It shows enhanced performance with improved packet delivery ratio, reduced latency and has least energy dissipation than other SI algorithms. It is suitable for real network deployments. In future, they want to verify the scalability of the proposed BeeSensor protocol on very large scale WSNs and validate it on real testbed of WSNs.

The Bee-Sensor-C routing protocol (Cai et al., 2015) is based on BeeSensor (a bee-inspired routing system) that can dynamically create clusters and transmit data in parallel. Using fractional calculus and the ABC method, a cluster-based routing protocol (Kumar & Kumar, 2016b) is developed. By selecting the CH optimally, the major goal was to optimize the network’s residual energy and longevity. Energy expenditure, distance, and latency were used to define the fitness function.

In WSN, a few researchers such as Arora, Sharma & Sachdeva (2020, 2019) used a heuristic ACO-based self-organized energy-balanced algorithm. Cluster formation, multipath generation, and data transfer are the three stages of the approach. The appropriate number of sensor nodes is chosen as a CH in cluster creation, and the other nodes join the nearest CH to build the cluster. Using the ACO algorithm, several routes between CH and cluster member nodes are investigated. For data transmission between cluster members and CH nodes, ACO chooses an energy-efficient optimal path. Because the above-mentioned routing system has a low convergence rate, next-hop selection is ineffective.

Ari et al. (2016) introduced ABC-SD, a centralized clustering based routing approach using ABC metaheuristic. A multi-objective fitness function is used to choose CHs, as well as a cost-based routing function makes a trade-off between the hops count in the route and energy efficiency.

Quantum version of the ABC algorithm as provided by Wang, Chen & Dong (2016) integrated quantum computing into the ABC algorithm, and introduced advanced quantum ABC algorithm. This improved global search capability with beneficial convergence. The scheme is applied to WSN route optimization to increase network performance and reliability. The clustering algorithm is then built on the basis of the quantum ABC algorithm aiming at uneven load in the WSN clustering phase without taking into account nodes residual energy, node intensity and node locations.

Mann & Singh (2017a) proposed a BeeSwarm protocol including three phases: BeeCluster building, BeeSearch for route discovery, and BeeCarrier for data transfer to enhance the routing process in WSN. BeeCluster, BeeSearch, and BeeCarrier contribute to the protocol’s robustness. The cluster is created by running the CHs selection and creation method. During the setup phase, the CH is initially chosen using the ABC meta-heuristic. The creation of clusters begins when CH has been chosen. To establish clusters, a join request is sent to all the nearby nodes from the CH. The BeeSearch phase establishes communication links *via* scout bees. Forward and backward search techniques are used to accomplish this. The forward search is used to investigate the network. The backward search method creates and maintains a route between multiple nodes and BS.

In the work by Mann & Singh (2017b), an improved ABC (iABC) algorithm is presented to optimize energy consumption of a network. In this approach, an improved solution search equation is proposed to find optimal solution for enhancing its exploitation function and then an enhanced population sampling scheme called Student’s-t distribution technique that helps in enhancing global convergence is used. To further enhance the capabilities of this approach, ABC based cluster formation method called BeeCluster is presented to select optimal CH and to improve energy-efficiency of the system. Beecluster is a clustering protocol that will improve the convergence speed of the ABC algorithm. The results indicate better WSN lifetime, energy utilization, throughput, packet delivery ratio and latency.

A distributed swarm ABC (DSABC) methodology is developed by Panda (2018) to enhance the dynamics of CHs and normal nodes in WSNs. It can reduce node energy loss and balance the network’s interference-aware energy usage. DSABC improves network performance with fewer control parameters in its objective function, making it easy to apply in clustered WSNs.

The work by Mann & Singh (2019) also aimed to improve the routing and energy efficiency of WSN. To increase its exploitation capabilities, they suggested an enhanced ABC metaheuristic with an improved search equation. To improve the the global convergence, an improved population sampling approach is added through the Student’s-t distribution that needs only a single control parameter to determine and save, therefore increasing the efficiency of the current ABC method. Furthermore, an energy-efficient bee clustering method is given based on the proposed WSN metaheuristic, which chooses optimum CHs using an effective fitness and enhanced search equation.

A chaotic discrete ABC algorithm (CDABCP) presented by Masdari, Barshande & Ozdemir (2019) to form multiple cluster levels in WSN, with the objective of reducing WSN energy consumption. Based on an enhanced ABC algorithm, the work by Zhang et al. (2020) developed a seamless clustered multi-hop routing technique (IABCP). Routing pathways can only be created using crude approaches due to the limited sensing range and intelligence of normal nodes. The sink node’s movement will use a significant quantity of energy. The sink node is given the responsibility of generating the routing table in order to address this challenge. The sink node will use the enhanced ABC method to build the routing table. Furthermore, a new technique for selecting CH nodes is used; each node calculates the claimed CH time using the average energy of the adjacent nodes and its own leftover energy. They also included a CH-*β* node as a sub CH. When the CH node has completed the required number of substitution rounds, the CH role is shifted to the CH-*β* node.

### Solutions based Evolutionary Computation and Trajectory

A differential evolution based clustering algorithm (DECA) is introduced by Kuila & Jana (2014) to prolong WSN lifetime and to prevent faster death of CH. They used special nodes known as relay nodes or gateways with extra amount of energy. These are also battery operated and act as a CH. So, in some cases if gateways are not provided with accurate number of sensor nodes, they may get overloaded and result in death of gateways and affect the overall network performance. To avoid this, proper allocation of nodes to the gateways must be done. The main focus of this approach is to extend WSN lifetime by considering the power usage of nodes and gateways (*i.e*., CH). In this approach, they use local improvement in traditional DE which helps the solution to converge faster. An efficient vector encoding scheme is proposed in this for complete clustering solution. This clustering approach has two phases: (1) In bootstrapping phase, all gateways and nodes are assigned unique IDs which are broadcasted using MAC layer protocol. The IDs of their nodes are collected by the gateways in their communication range and sent to BS. (2) Then in clustering phase, clustering algorithm is executed. When cluster formation is completed, all gateways broadcast their unique IDs to their member nodes using one hop communication. They also derived efficient fitness function which includes the energy expended by both the nodes and the gateways to extend WSN lifetime. The results show that the method provides better clustering and extends WSN lifetime.

Some authors also worked in applying metaheuristic local search method such as Tabu search (TS) which was primarily used for mathematical optimization problems. TS enhances the performance of local search by prohibiting already visited solutions or others through user-provided rules. Orojloo & Haghighat (2016) developed a Tabu search based routing (TSRA) to establish optimal routing path in WSNs. It uses a strategy for neighborhood solutions search and move or next-hop selection using Tabu search, which leads to attain better results for different WSNs. It combines energy consumed and hop counts to make better routing decision. This contributes to balanced data transmission with reduced energy dissipation and routing cost. For different randomly generated networks, results show that the TSRA obtains more balanced transmission between the nodes, decreases energy usage and routing costs, and increases the life of the WSN.

GA with low computational cost can not only work directly on objects, automatically accomplish and result an optimal search space, and also adaptively modify the search path, converge quickly, and eventually find the best global solution by simulating the evolution of biological populations in nature. As a result, they were often employed in clustering routing from CHs selection to route search or hybrid by Kong et al. (2018), Ragavan & Ramasamy (2018), Al-Shalabi et al. (2019), Wang et al. (2018), Wang et al. (2020). The key things to consider while constructing GAs are population initiation, fitness function specification, GA operators, and determination conditions.

### Solutions based physical computations, nature inspired, and learning

Optics inspired optimization (OIO) is a meta-heuristic optimization technique applied to address various NP-Hard problems. Lalwani, Banka & Kumar (2017) treated cluster formation and routing in WSN as OIO and proposed algorithm named CRWO. The CH selection process is formulated using OIO by considering energy, node degree and distance as main parameters. Moreover, an OIO based method is developed for routing which determines the path connecting CH to BS.

Morsy, AbdelHay & Kishk (2018) presented an energy efficient clustering and routing technique using gravitational search algorithm (GSA). A single-objective optimization problem is used to structure the CH selection procedure. This assist in determining the best collection of CHs to construct one-hop clusters that are energy efficient. The suggested method’s scalability and stability are improved by using the GSA algorithm.

Multi-objective nature-inspired algorithm based on shuffled frog-leaping algorithm and firefly algorithm (named MOSFA) is proposed by Barzin et al. (2020). This is a cluster based and adaptive routing scheme based on multi-hop path. Multi-objective function of MOSFA with respect to various parameters (*e.g*., distances (intra and inter cluster), the nodes’ remaining energy, overlap, distances to sink, and cluster load) is utilized to choose suitable CHs per round. Also, for the identification of forwarder nodes in the routing stage, another multi-objective function is provided. In both the clustering and multi-hop stages, the controllable parameters of MOSFA is being adaptively tuned to deliver the optimal performance with respect to application and network specifications.

Zeng & Dong (2016) offered an improved harmony search method for developing an energy-efficient routing scheme for WSN. The goal function is evaluated using the route length and power usage in this algorithm. This approach reduces energy usage while also extending the network’s lifespan.

A cluster based routing scheme using harmony search algorithm (HSA) to enhance the energy efficiency of WSN through two phases, (a) clustering and (b) routing, is proposed by Zeng et al. (2017). The approach is based on many enhancements to the HS algorithm, including the introduction of a discrete harmony encoding scheme for clustering as well as a local search strategy to identify the optimal harmony inside the harmony memory during iterations. For balancing the gateway energy consumption throughout the routing process, the enhanced HS based energy-efficient routing algorithm by Zeng & Dong (2016) is used.

The routing technique by Zeng et al. (2017) is designed in a somewhat different way from Zeng & Dong (2016). The BS transmits the packet containing each gateway’s forwarding path to each gateway *via* multiple communication in reverse order of gateways inside the forwarding path at regular intervals. The routing algorithm may substantially balance the energy consumption of gateways in the transmission stage.

Lalwani et al. (2018) presented a HSA based CH decision scheme by taking distance, energy, and degree of nodes as input of the fitness function, in contrast to Zeng & Dong (2016) where, by considering distance only, non-CH nodes join the CH, which can induce imbalance in the CHs’ load and can cause fast depletion of network’s energy.

HSA based load balanced and energy-efficient clustering approach (HSCA-2) with multiple objectives is presented by Singh & Sharma (2018). The new condition of average distance was applied in this approach for better HSA convergence while initializing the harmony memory and its improvisation in random search as well as pitch adjustment. HSA is used by Poonguzhali & Ananthamoorthy (2020) for path maintenance and minimum density cluster selection in the WSN. This assists in providing the shortest path from the source to destination.

Bat algorithm (BA) has proven to be an effective optimization option for clustering by various works in literature (Cui et al., 2019; Nguyen, Pan & Kien, 2019; Cai et al., 2020). The compact bat algorithm (cBA), introduced by Nguyen, Pan & Kien (2019), is an optimization technique for a class of optimization issues involving devices with restricted hardware resources. For the probabilistic processes that produce each candidate for the solution of the cBA optimization, a real-valued prototype vector is utilized. The suggested cBA is thoroughly tested on a variety of continuous multimodal functions as well as situations involving uneven clustering of WSN (uWSN).

A version of the BA integrated with the centroid technique is implemented by Cui et al. (2019). For six distinct models, three different centroid methods were used. Also included is the velocity inertia-free updating equation. CEC2013 by Li et al. (2013) benchmarks in such schemes are used to compare optimization performance to conventional BA. The BA with weighted harmonic centroid (WHCBA) approach outperforms other algorithms in simulations. A two-stage CH selection method is created by incorporating WHCBA into the LEACH protocol, which can save more energy than the actual LEACH. A unified heuristic bat algorithm (UHBA) is suggested by Cai et al. (2020) to enhance the selection of CHs. This algorithm ensures that the selection of CHs will freely transform both global as well as local search.

Energy consumption and delay in relaying data to the sink are the two extremely important issues in WSNs with large number of deployed nodes. As a solution, the work by Mehmood et al. (2020) proposes an energy efficient, robust routing technique using ANN. The technique is termed as ELDC. In this scheme, training of the network is done using a huge data set which incorporates almost all scenarios of network. This helps to create a more reliable and adaptive network. Moreover, it utilizes the benefits of group based protocols to enhance the overall network lifetime. The use of ANN helps in yielding efficient threshold values for group’s chief node and CH selection based on back propagation and allows efficient, robust and intelligent group organization. A deep reinforcement learning (DRL) based traffic-control system is introduced by Lu et al. (2019), which considers traffic control as the prime strategy for learning process to enhancing energy efficiency. The system employs deep neural network for learning and provides the optimal routing path as output.

Künzel et al. (2020) introduced a Q-learning reliable routing method using a weighted agent strategy. They proposed a system in which an agent modifies the weights of a state-of-the-art graph-routing scheme. During network operation, the states of the agent reflect sets of weights, and the actions modify the weights. When the average network latency reduces or the predicted network lifespan improves, the agent is rewarded.

To solve the energy depletion problem in IoT based WSN, Dohare & Singh (2020) proposes an energy balanced integrated atom search optimization and electromagnetic force optimization (iASEF). To accomplish energy balanced clustering, atom search optimization is used to correctly equalize the energy usage of all sensor nodes. They provided answers to the CH selection linear programming problem as well as the inter-cluster routing issue. Atom search optimization was used to solve the linear problem of CH selection, and electromagnetism force optimization was used to address the inter-cluster routing problem by determining the next hop for data forwarding to BS.

### Solutions based Fuzzy Logic

In Sert, Bagci & Yazici (2015), a fuzzy based multi-objective and distributed method is proposed to perform effective WSN clustering. This multi-objective clustering solution aims to solve the energy hole and hot spots problems in stationary as well as evolving WSNs. A balance in energy dissipation is achieved by suitably tuning the cluster radius based on three parameters. Another fuzzy-based method of clustering is introduced by Akila & Venkatesan (2016) to determine the cooperative node (CN) that enters a cluster and uses PSO to construct the communication route between a CN and CH and uses PSO to construct the communication route between a CN and CH.

In Shokouhifar & Jalali (2017), an adaptive fuzzy based clustering technique for WSN is proposed (LEACH-SF). The proposed LEACH-SF protocol utilizes Fuzzy C-Means to form clusters and then suitable CHs are selected using Sugeno FIS with inputs: distance from cluster centroid, residual energy, distance to sink. Rather than updating the fuzzy rule base table manually, they used artificial bee colony algorithm (ABC) for suitable tuning of the fuzzy rules. The fitness function is formulated so as to improve the WSN lifetime according to application requirements.

In Zhang et al. (2018), adaptive green and reliable routing scheme based on FL is provided and that considers end-to-end transport delay, energy efficiency and transmission reliability. A fuzzy clustering scheme was presented by Hai, Son & Vinh (2017) to obtain the clustering results with the help of a mathematical model that considers 3D energy functions which are suitable for use in 3D WSNs. In Hamzah et al. (2019), a cluster based and energy efficient routing method called FL-EEC/D is proposed which uses the efficiency of FL to perform CH selection in cluster. The proposed FL based clustering scheme utilizes five major parameters in selecting the CH which includes node’s remaining energy, compactness, distance towards BS, density and average local energy consumption. Energy efficiency in terms of algorithm ability in achieving balanced energy consumption is measured using Gini index. The evaluation of simulation results in comparison with other algorithms indicate that the technique has enhanced the energy efficiency and WSN lifetime.

Another fuzzy based technique proposed by Razzaq & Shin (2019) utilizes weighted sum approach in which a node’s weight within a cluster is calculated and compared with basic cluster weight to select the appropriate CH. The node whose weight is nearest to the cluster’s standard weight will be selected as CH. This helps to achieve better load distribution. They proposed a data routing algorithm which uses a weight function based on intra-cluster communication cost and fuzzy membership function to assign weight to each link. Then, Dijkstra’s algorithm is adopted to determine best (minimum weight) route. The proposed technique is energy efficient and experiments show that it achieves better results in terms of throughput, WSN lifetime and energy consumption.

In Yuste-Delgado, Cuevas-Martinez & Triviño-Cabrera (2019), a distributed algorithm for unequal clustering in WSNs that employs a new set of input variables is presented. In comparison to earlier studies, these factors aid in a more reliable assessment of a node’s functioning as a CH. The proposed technique uses local node data to decrease data exchange, interruption, and transmission process consumption. The suggested clustering technique is based on the type-2 FL system, whose knowledge base is sampled to allow it to be implemented in a node, due to the inherent uncertainty of the local data. An unequal cluster size scheme is employed in particular since it has been found that these techniques are more suited for energy efficiency.

In Kiran, Smys & Bindhu (2020) fuzzy-based approach for clustering is adopted, which selects an optimal CH. Using energy and distance as fuzzy descriptors, fuzzy clustering is accomplished. However, in many situations, fuzzy-based clustering is considered to be inefficient where the WSN is heterogeneous. To improve performance, a multidirectional routing scheme is presented, along with fuzzy clustering. With this, it is possible to find multiple routes between the node and BS and the least hop path among them will then be selected for routing. Sreedharan & Pete (2020) presents a fuzzy multi-criteria decision making (MCDM)-based CH (CH) selection and hybrid routing protocol to solve expendability, cost, maintenance, availability of software, and performance characteristics issues. The work by Verma et al. (2020) presented a FL based approach for effective clustering (FLEC) in homogeneous WSNs with MS. FLEC resolves the limitations of LEACH-Fuzzy (Nayak & Devulapalli, 2015) by using the idea of average threshold and average energy based probability for selection of appropriate CHs. In addition, average CH energy has been incorporated in Fuzzy descriptors to determine super CH nodes.

### Solutions based hybrid methods

A hybrid HSA and PSO algorithm is proposed for energy-efficient CH selection (Shankar, Shanmugavel & Rajesh, 2016). The scheme is successful in achieving a faster convergence in global search. The proposed algorithm exhibits high HSA search efficiency and dynamic PSO capability that enhances the WSN lifetime.

The PSO-HSA approach is utilized, where PSO is used to maximize the selection of CH, and HSA (harmony search algorithm) is adopted to select the best path by Anand & Pandey (2017). Therefore, the PSO-HSA approach is effective since, by integrating the advantages of both PSO and HSA, it chooses the best clusters and path.

The results of the study described by Sindhuja & Ramamoorthy (2017) show that their proposed bacterial foraging optimization (BFO)-fuzzy technique gives better results when compared to destination-sequenced distance vector (DSDV).

Multipath routing helps to create multiple paths for data transmission from source and destination. The use of multipath routing can therefore enhance the WSN performance through its efficiency in determining alternate paths between source and sink. The path reliability is checked using a fuzzy approach that has limited rules (FMLR) by Sindhuja & Ramamoorthy (2017). The method assess the reliability of a path in terms of multiple parameters. The proper tuning of parameters helps to achieve optimization of membership function.

In Tomar & Shukla (2019), the authors proposed hop count based energy efficient routing scheme for WSN using gravitational search algorithm (GSA) and fuzzy based clustering. CH selection is done using GSA, and based on its weight, the nodes join the CH to form cluster. Then, a super CH (SCH) is selected among the CHs using fuzzy inference system. It is the duty of the selected SCH to collect and forward the CH’s data packets to the sink (BS). The data transmission route is selected based on the hop count of nodes to enhance the efficiency.

To improve the network stability, it is essential to have a balanced routing scheme. In Mina et al. (2020), an enhanced fuzzy based flower pollination algorithm for energy-efficient clustering is proposed to improve the network stability.

Hybrid swarm by Kumar & Kumar (2016a) provide a combination of ABC and ACO algorithms for power efficient routing in hierarchical clustered WSN.

To solve the WSN routing challenge, the work by Fanian & Rafsanjani (2020) used the shuffled frog leaping algorithm (SFLA) and proposed a fuzzy based multi-hop cluster formation algorithm (FMSFLA). SFLA is adopted to effectively configure and optimize the fuzzy rule-base table, as well as used five adjustable parameters in both CH and parent selection phases, according to application requirements. The method considers multiple effective parameters including distance towards BS, count of neighboring nodes, energy, real distances of nodes from BS, average load of path, overlap and delay, and the hot-spots issue, to meet the required performance. FMSFLA operates in rounds. Selection of CH and parent, cluster creation, and steady state processes are executed in each round. During the CH election process, the fuzzy output and energy threshold and the overlap rate of adjacent CHs is considered to achieve better selection. The process of selecting parent start by finding the levels of CHs in the WSN, and the parent of each CH is determined in terms of fuzzy output with respect to the application scenario. Then the cluster creation process is executed. In the final steady state phase, the information gathered by CHs is transmitted to the BS through their parents.

## Discussions

**Figure 4:**
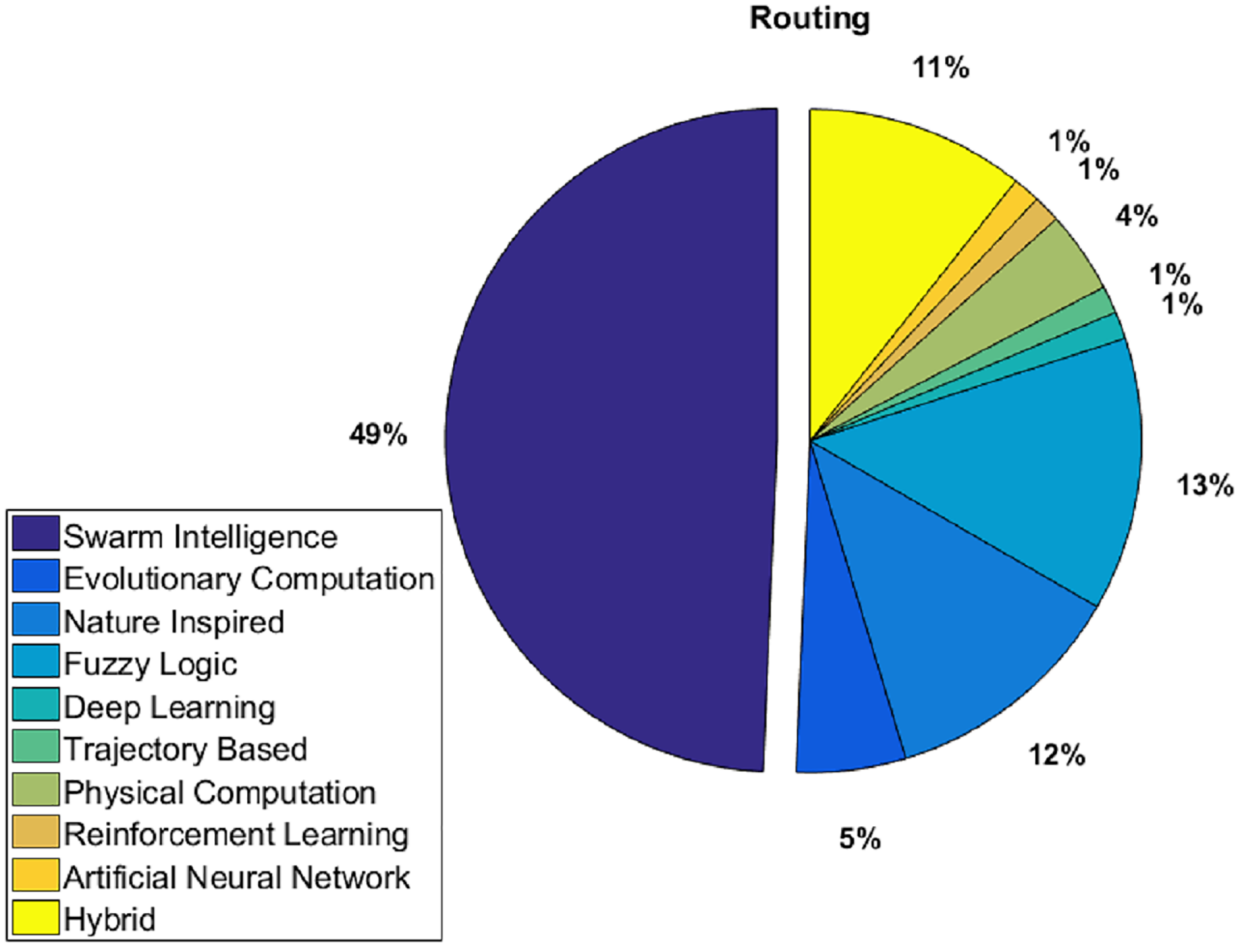
AI methods with respect to routing challenge in WSN.

From the findings depicted in Fig. 4, 49% articles applied SI, 12% applied nature inspired, 13% applied FL, 5% applied evolutionary computation and 11% applied hybrid and 10% applied other methods (see Fig. 4).

Some AI algorithms have been used frequently to solve the WSN routing challenge. GA, PSO, ACO, ABC, BA, DL and fuzzy have been used more than other algorithms by the research community to deal with WSN routing challenge. These algorithms are selected based on problem nature or algorithm features. If problem parameters have fuzzy feature and membership function, FL method is suitable to deal with this problem. When problem size is too vast, and needs to be adapted to a particular case and with respect to time, we need to solve it using DL. For *e.g*., in intrusion detection, online detection of network attacks, a good quality feature representation for clustering and enhance the classification performance. GA is very efficient and stable in exploring the search space for global optimal solutions. It helps to solve general, unconstrained and bound constrained optimization problems, and it performs very well for large scale optimization problems. Both continuous and discrete parameters can be used by Bajpai & Kumar (2010), El-Sawy et al. (2014), Abdmouleh et al. (2017). So researchers used GA for these characteristics that help to solve a wide variety of WSN challenges.

From Fig. 4 and Table 3, we can notice that SI algorithms are used abundantly in handling the WSN routing problems. One of the commonly adapted algorithm is PSO. This is because PSO has several key advantages over other optimization techniques. PSO is a derivative-free algorithm. It has the flexibility to be integrated with other optimization techniques. It is less sensitive to the nature of the objective function and can handle objective functions with stochastic nature. PSO has few parameters, able to run parallel computation, can converge fast and is easy to implement (Abdmouleh et al., 2017).

ACO algorithm is based on ants where their task is to search for food. Each ant takes a path in searching. That makes ACO suitable to work in parallel problems and is applicable to solve the routing challenge. ACO has many characteristics that encourage researchers for usage. It can adapt to changes, have guaranteed convergence and can provide good solutions rapidly (Abdmouleh et al., 2017).

Working of ABC is focused on the behavior of bees in visiting and selecting best source of food (flowers). So, it works well in selection problems such as choosing best CHs in WSN for data routing. ABC is easy to use, available for hybridization with other algorithms (Abdmouleh et al., 2017). Like many meta-heuristic algorithms, flexibility and simplicity are the main highlights of Bat algorithm (BA). At the same time, BA possess other advantages over other metaheuristic algorithms. BA has automatic zooming (into the area where promising solution can be found) and parameter control (tuning parameters over iterations) capabilities which allows BA to jump automatically from exploration to exploitation when optimal solution is approaching, thus provide fast convergence (Yang, 2013). For these reasons, BA is becoming increasing popular for solving routing challenge such as determining optimal CHs. The harmony search (HS) approach is driven by the fundamental concepts of musician improvisation of harmony. HS algorithm has the distinctive characteristics of flexibility and search performance that have made it better to deal with a variety of challenging problems over the past decade. In the last decade, many revised HS schemes have been investigated in order to improve the efficiency of the original version (Gao et al., 2015). For this reason, some recent research works in WSN use a combination of HS with another algorithm to boost the efficiency of WSNs.

As we have discussed above, population based meta-heuristic algorithms are used extensively because of their efficiency in providing best results. Their individuals don’t use any symbolic reasoning and don’t maintain a plan about the future, both of which are computationally expensive. Moreover, these techniques involve only limited memory requirements as they don’t need to keep a lot of previous information. It can be robust, and maintain good quality performance in rapidly changing and diverse environments.

As we notice from Fig. 4 and Table 3, some AI methods such as MAS and RL have been applied rarely by research community to WSNs. MAS has many challenges when it is used, which includes coordination between agents (including consensus, controllability, synchronization, connectivity, and formation), learning, fault detection, task allocation, localization, organization, and security. The difficulty in overcoming these challenges when the MAS is applied to solve a problem makes MAS rarely used by researchers (Dorri, Kanhere & Jurdak, 2018). The deep reinforcement learning is also used rarely in WSN. This is because DRL faces main challenges like non-stationary environment, partial observability of the environment, continuous action spaces and is computationally expensive (Nguyen, Nguyen & Nahavandi, 2018).

Table 4 shows a general comparison of utilized AI methods and their capabilities with respect to computational requirements, memory requirements, and flexibility (Kulkarni, Forster & Venayagamoorthy, 2010; Yang, 2012; Kar, 2016; Darwish, 2018; Renman & Fristedt, 2015; Henderson, Jacobson & Johnson, 2003; Sabri, Puteh & Mahmood, 2013; Thompson et al., 2020; Chen et al., 2020; Siebers & Aickelin, 2008; Salazar et al., 2018). Computational requirements and memory requirements are classified into high, medium or low, which explain AI methods that have high and medium class capabilities could be suitable for running in a centralized manner (at BS side), while AI methods that have low class capabilities could be suitable for executing in a distributed manner (at node side). Flexibility is classified into high or low and it is the capability of the AI based algorithms to conform with environmental changes (Kulkarni, Forster & Venayagamoorthy, 2010). As shown in Table 4, most of the AI methods are flexible for changes as they can be executed in a flexible manner upon occurrence of changes. Moreover, AI methods have the ability to find the best solution for some problems. For best performance, the designer should correctly select a method or combination according to the exact scenario to meet the needs of the application. Simulation is an effective method of determining whether WSNs are appropriate before they are deployed. Simulation can assesses, for instance, the scalability of algorithms without being constrained by a hardware platform. In addition, simulators simplify WSN application development. Therefore, simulation is a powerful research tool used by the majority of researchers. In regards to the summarised research articles in Table 3, the majority of the authors utilized simulation in their research. MATLAB, NS-2, OPNET, and OMNet++ are some of the most widely used simulators.

**Table 4:** General comparison of AI methods utilized in WSN.

AI method	Comptational requirements	Memory requirements	Flexiblilty
ANN	Medium	Medium	Low
Fuzzy Logic	Medium	Medium	High
Evolution Computation	Medium	High	Low
SI	Low	Medium	High
RL	Low	Medium	High
Trajectory Based	High	High	High
Nature Inspired	Low	Medium	High
Physical Computations	High	Low	High
Deep Learning	High	High	Low
Multi-Agent Systems	High	Low	High

## Future scope

In this section, we identify the promising research directions in applying AI-based solutions, with the aim to promote and facilitate further research. Algorithms like iASEF (Dohare & Singh, 2020) should be used in the future to solve physical world issues. Furthermore, novel optimization strategies based on self-adaptive methodologies may be used to address the network’s energy issue. The scalability of the existing schemes can be tested on very large-scale sensor networks and also tests can be done to evaluate the performance on a real test-bed of sensor networks with the help of simulation testing tools like TOSSIM. Different network scenarios (or dynamic scenarios) can be evaluated on a real test bed of sensor devices within a specified application area. The trade-off among the performance factors can be emphasized in modeling of such networks.

Additionally, by deploying on real WSN hardware, the performance can be evaluated, and current techniques may be enhanced based on the knowledge gained from the real-time validation and verification. Moreover, the routing schemes should be tested precisely for its QoS metrics to validate its effectiveness on a real WSN. For testing and increasing the scalability factor, several WSN layouts can be used. Future study might include the deliberate mobility of sensor nodes, gateways, and BSs to assess the impact of changing their location. MANETs or VANETs might be used to improve this in the future. The scenario of changing packet length can also be investigated further.

More study is needed in the future on strategies for maintaining a continuous alternate path and increasing the path’s dependability in one-way links, which are common in WSN environments. Furthermore, certain path enhancements may be performed, such as comparing the outcomes of previous efforts with other techniques (*e.g*., recursive neural networks). In this way, the existing work can be applied, and adapted if necessary, to actual network environments with heterogeneous WSNs. This can support in enhancing the performance for IoT based big data sensing and management systems.

Future research initiatives can be motivated by the presented results and expanded to allow two-hop hierarchical clusters with a link quality threshold between any cluster member and its following hop, resulting in higher-quality clusters that maximize throughput. Future work can utilize adaptive power control method to analyze the enhancement in the network energy efficiency. In fuzzy based methods, designing membership functions, arranging the inference rules and their influence on system performance are worth further research studies. Computational cost of sink can be reduced by employing different multi-objective algorithms. The need of location information of nodes can be eliminated by estimating energy consumption of nodes while communicating to each other. Different methods can be considered for multi-hop routing between nodes to CHs, and to divide the observed area into sub areas for reducing computational cost and the updating the clustering scheme for heterogeneous nodes have to be investigated further to cope with large topological areas.

One possible future enhancement might be to optimize the combined routing algorithm and the sleep scheduling method for WSN lifespan maximization, making heterogeneous WSNs more broadly applicable. Another future research is in the direction of designing metaheuristic schemes for clustering and data routing with permanent CHs in addition to considering metaheuristic node deployment approaches for enhancing the coverage with reduced count of deployed nodes. Furthermore, most works do not account for the failure of any node or CH, necessitating the development of fault-tolerant algorithms for dynamically changing network configurations in the future. As a result, while creating WSNs, researchers may concentrate more on fault-tolerance and time-sensitive scenarios.

Automatic strategies to tune the controllable parameters of the optimization algorithms and solving multi-objective routing problems in emerging IoT based dense WSN, software defined networking (SDN), information centric networks (ICN) and vehicular adhoc networks (VANETs) require further attention. It is also necessary to study further how to estimate and reduce the required learning and exploration duration, as well as how to quantify the predicted performance gains. These problems might be solved by creating a simulation/optimization module that uses simulation or actual data to offer information on network performance as well as an optimal configuration to employ.

Other research options include developing Q-Learning reliable routing (QLRR) approaches that can build routes while taking into account transmission power, mobility, coexistence, scheduling, and real-time requirements; simulation and experiments using other centralized network protocols; and evaluating state-of-the-art routing strategies in real Industrial WSNs (IWSNs), physical channel conditions and specific IWSN applications.

Nowadays, we are witnessing a growth in the number of AI-based systems and solutions which facilitate the optimization of services in the field of WSN. The combination of both AI methods and WSNs have now become a reality, offering benefits to the area of IoT, and allow systems to learn and to monitor activities and support the decision-making process. From analysis, we have noticed that the research community is focused highly on routing and clustering challenge. Moreover, the most appropriate AI methods used by the research community is SI, where 49% of research articles applied SI methods, while other AI methods gained less attention from research community, which is due to problem nature or method characteristics.

For enhancement of WSN, new AI algorithms as well as different strategies to embed these algorithms in WSNs have to be encouraged in future. At present, the majority of the solutions presented here applied AI methods to limited problems in some areas. Most problems stem from incompatibility between layers and high human interaction. Self-adaptivity is required for setting and adjustment of solutions. Hybrid approaches that optimize the resource utilization in WSN need to be developed (Jabbar et al., 2013). Learning platforms and prototypes are needed rather than specialized solutions.

In future, further research to devise efficient distributed data mining solution for WSN with improvements in noisy data filtering process have to be encouraged. The layered structure and other typical features of DNN makes it a favorable option for application in such scenarios. The main challenges include training of distributed multi-layered DNN and better tradeoff between power consumption during processing and transmission. A further interesting area of study for AI methods in WSNs is known to be parameter learning and optimization.

The application of AI optimization methods to overcome the challenges of MWSNs is a potential future direction. Some research challenges still remain relatively unresolved which include transmission delay, balancing the power consumption, reliability and safety of MWSNs. A combination of SI with other optimization techniques have to be encouraged in future. The cross-layer optimization model challenges must to be treated in a better way during the optimization of MWSNs. The results from the study of human-related biological features can be incorporated in future for further improvement of solutions to such problems. Distributed and real time application of algorithms in light weight form could be a future research direction to solve the challenges of dynamic MWSNs in future.

As the study shows, most of the existing solutions based on AI are only simulation based. The AI techniques should be implemented and analyzed in real-time environment (Sarobin & Ganesan, 2015; Montoya, Restrepo & Ovalle, 2010). This should be encouraged in future. More investigations are required to show how AI methods can be adapted. Cross-layer approaches using AI methods are rarely applied to challenges and is still a vital open research area. Also, hybrid AI methods are less applied and need to be discussed deeply. It can be expected that the future research will likely consider heterogeneity, dynamic environments and varying communication constraints during algorithms design. The application of AI techniques make the WSN cognitive towards managing and overcoming the challenges which arise during operation. We hope that the concepts provided in this article direct the researchers for the use of AI in solving the challenging WSN routing issues by making the nodes more intelligent.

## Conclusion

In this article, we have provided a comprehensive review of routing problem in WSNs. Various AI methods in WSNs are briefly introduced along with their classifications. The AI techniques which are used by researchers to address the routing challenge in WSNs are briefly explained for the span of 2010 to 2020. About 75 relevant research journal articles indexed in a reliable database sources have been discussed and summarized. The discussions show that some AI methods have been used frequently to solve the WSN routing challenge. SI has been used more than other methods by the research community to deal with WSN routing challenge, where SI constitutes 49% of the total. Some AI methods such as multi-agent system and reinforcement learning have been applied rarely by research community to WSNs. While other method such as hybrid methods need more attention from the research community. An application designer should choose the right method or combination of methods based on the exact scenario to produce the best results. For that, we present a general comparison of utilized AI methods and their capabilities with respect to computational requirements, memory requirements, and flexibility that could help application designers. Where, computational requirements and memory requirements are classified into high, medium, or low, that is to show which AI methods could be suitable for running in a centralized manner (that may have high and medium class capabilities), and which methods could be suitable to work in a distributed manner (that may have low class capabilities). Finally, promising future research directions in applying AI-based solutions toward solving routing challenge in WSNs, with the aim to promote and facilitate further research are identified.

## Additional Information and Declarations

### Data Availability

The following information was supplied regarding data availability: This is a review article, there is no code or data.
